# Machine learning algorithms for predicting low birth weight in Ethiopia

**DOI:** 10.1186/s12911-022-01981-9

**Published:** 2022-09-05

**Authors:** Wondesen Teshome Bekele

**Affiliations:** Department of Statistics, College of Natural and Computational Sciences, Dire Dawa University, Dire Dawa, Ethiopia

**Keywords:** Classification, Low-birth-weight, EDHS, Ethiopia, XGB, GB, RF

## Abstract

**Background:**

Birth weight is a significant determinant of the likelihood of survival of an infant. Babies born at low birth weight are 25 times more likely to die than at normal birth weight. Low birth weight (LBW) affects one out of every seven newborns, accounting for about 14.6 percent of the babies born worldwide. Moreover, the prevalence of LBW varies substantially by region, with 7.2 per cent in the developed regions and 13.7 per cent in Africa, respectively. Ethiopia has a large burden of LBW, around half of Africa. These newborns were more likely to die within the first month of birth or to have long-term implications. These are stunted growth, low IQ, overweight or obesity, developing heart disease, diabetes, and early death. Therefore, the ability to predict the LBW is the better preventive measure and indicator of infant health risks.

**Method:**

This study implemented predictive LBW models based on the data obtained from the Ethiopia Demographic and Health Survey 2016. This study was employed to compare and identify the best-suited classifier for predictive classification among Logistic Regression, Decision Tree, Naive Bayes, K-Nearest Neighbor, Random Forest (RF), Support Vector Machine, Gradient Boosting, and Extreme Gradient Boosting.

**Results:**

Data preprocessing is conducted, including data cleaning. The Normal and LBW are the binary target category in this study. The study reveals that RF was the best classifier and predicts LBW with 91.60 percent accuracy, 91.60 percent Recall, 96.80 percent ROC-AUC, 91.60 percent F1 Score, 1.05 percent Hamming loss, and 81.86 percent Jaccard score.

**Conclusion:**

The RF predicted the occurrence of LBW more accurately and effectively than other classifiers in Ethiopia Demographic Health Survey. Gender of the child, marriage to birth interval, mother’s occupation and mother’s age were Ethiopia’s top four critical predictors of low birth weight in Ethiopia.

**Supplementary Information:**

The online version contains supplementary material available at 10.1186/s12911-022-01981-9.

## Background

Birth weight has a significant influence on its chances of survival. Low birth weight (LBW) is becoming more an issue, particularly in emerging countries. A major cause of neonatal death is low birth weight, less than 2500 g [1]. Babies born at a low birth weight are 25 times more likely to die than babies born at a normal birth weight [2]. It's also a good indicator of a child's future health complications. Low birth weight affects one out of every seven newborns, accounting for about 14.6 percent of the babies born worldwide. The prevalence varies substantially by region, with rates of 7.2 per cent in the More Developed Regions, 13.7 per cent in Africa, and 17.3 per cent in Asia, respectively. These newborns were more likely to die within the first month of birth or to have long-term implications. These are stunted growth, low IQ, overweight or obesity, developing heart disease, diabetes, and early death [1, 3].

Evidence shows that the global prevalence of LBW dropped by 1.2 percent each year between 2000 and 2015, meaning that progress is insufficient to fulfil the World Health Assembly's low birth weight target of 30 percent by 2025 [1]. LBW is still a serious public health concern across the world [1], putting neonates and newborns at an increased risk of death and morbidity. As a result, one of the main aims of the 'A World Fit for Children' initiative is to reduce low birth weight as a significant contribution to the Millennium Development Goal.

Low birth weight can be caused by a variety of circumstances, depending on the region [1, 2]. Low birth weight is associated with preterm in industrialized countries is caused by maternal age, smoking, multi-parity, and caesarean section. Low birth weight is caused by poor fetal growth is linked to poor maternal nutrition before and throughout pregnancy in less developed countries. Understanding the importance of preterm delivery and poor fetal growth as causes of low birth weight is critical for developing effective prevention interventions. Moreover, [4] revealed education was one of the predictors in LBW modeling.

Numerous studies have found that the most prevalent causes of low birth weight are prematurity and intrauterine growth restriction. In developed countries, the most common reason is preterm birth, but in underdeveloped countries, the most common cause is intrauterine growth restriction [5]. In addition, inadequate weight gain during pregnancy, low pre-pregnancy weight, short stature, the gender of the newborn (being female), hard physical labour during pregnancy, illness (especially infections), women’s lower socioeconomic status, and a lack of antenatal care (ANC) have been identified, as risk factors for low birth weight in developing countries [6]. Low birth weight is also associated with multiple gestations, young motherhood, genetic factors, poor maternal education, poor maternal nutrition before and during pregnancy, and poorer maternal anthropometric measurements [7, 8].

Even though LBW is a worldwide concern, the condition is most prevalent in the world's poorest areas, Sub-Saharan Africa and Southern Asia [9], suggesting that the disease has the strongest association with socioeconomic status [10, 11]. As a result, understanding the relationship between LBW and socioeconomic status helps to a strategy to address the problem more effectively in resource-constrained nations like Ethiopia. Women with lower socioeconomic status are more likely to have LBW children [12], although rising socioeconomic status may not necessarily result in better LBW reduction results.

According to [13], Ethiopia has a high prevalence of low birth weight variations. Newborn sex, prenatal care follow-up, pregnancy-induced hypertension, preterm birth, and mother's residency linked to LBW. Community-based health promotion initiatives on maternal nutrition during pregnancy, prenatal follow-up, and early treatment are needed to reduce the effects of LBW. Several studies in Ethiopia have been conducted to assess the prevalence and risk factors for low birth weight and have found that low birth weight ranges from 6 to 29.1%. Furthermore, low birth weight was associated with the infant’s sex, pregnancy-induced hypertension, ANC follow-up, preterm, parity, and residence [14–16].

Ethiopia has a birth weight burden of around half of the SSA, at around 11% [17]. As a result, understanding how to predict the LBW is beneficial and preventive. This study provides an LBW prediction model in Ethiopia. It also seeks to determine which classifier is best suited for predictive classification. The study used Logistic Regression (LR), Decision Tree (DT), Naive Bayes (NB), K-Nearest Neighbor (K-NN), Random Forest (RF), Support Vector Machine (SVM), Gradient Boosting (GB), and Extreme Gradient Boosting (XGB) [18–20].

## Methods

This study ML flows first displays the data source, pre-processing stage, feature selection for the classifier, hyper-parameter tuning, and classifier's functioning technique for the classification.

### Data source

The data of this study was obtained from the fourth wave of the Ethiopia Demographic and Health Survey, which took place in 2016 (EDHS-2016). The EDHS-2016 data collection period ran from January 18 to June 27, 2016. The survey was conducted under Ethiopia's Central Statistical Agency (CSA), with technical assistance supplied by ICF and financial support provided by other international organizations. The goal of the EDHS is to produce up-to-date estimates of the population's significant demographic and health variables [17]. The survey encompassed the country’s nine regions and two city administrations, generating data that was representative of the country as a whole and for urban and rural populations. The survey data was gathered using a stratified two-stage sampling procedure to choose a representative sample. There were 21 sample strata from 11 administrative states in all.

Separate enumeration areas (EAs) were chosen in two phases in each stratum. The EDHS-2016 selected 645 EAs at random from a total of 84 915 EAs in Ethiopia, with a probability proportional to EA size and independent selection in each stratum. The second stage of the sampling procedure involved picking a predetermined number of 28 households from each EA using an equal probability systematic approach [17]. There were 16,650 households in the EDHS-2016. Women aged 15–49 years and men aged 15–59 years old were eligible for the interview regardless of residence type. In all, 16,583 women aged 15–49 years were located in all examined houses, with 15,683 women accessible for an interview, resulting in a 95 percent response rate. To avoid bias, data collectors exclusively interviewed pre-selected households, and there were no replacements or adjustments to the pre-selected households during the implementation stages [17].

Low birth weight (LBW) is the study's target variable. Birth weight data were obtained in the survey for children born five years before the interview. There were 10,641 births in the five years before the survey. However, only 2110 newborns have birth weight data, accounting for around 14% (weighted) of the total number of births in the study.

### Preprocessing of data

This study employed data pre-processing to eliminate missing values, noisy data, and incompatible data since it is done in its raw form. Pre-processing, cleaning, integrating, transforming, reducing, and discretizing data are helpful [21]. In this study, like in other data mining methods, the initial stage of data pre-processing is necessary. Data cleaning, feature selection and resampling strategies are the three fundamental processes. EDHS initially contained 10,641 records that needed to be cleaned up (i.e. samples) (i.e. samples). The survey utilized to gather the data had a broad goal of satisfying all of Ethiopia's health-related needs. The LBW issue has a critical fault since the weight variable was missing in most samples (i.e. the weight of the infant was not recorded). Because the 'infant weight' feature is not available, 84 per cent of survey samples cannot be used and must be deleted. A large fraction of the 2,110 samples available had more than four or five missing data for the critical 25 features, comprising nearly 14% (weighted) of the sampled births in the survey.

Second, each sample in the dataset includes over a thousand features; therefore, feature selection is essential. A closer look reveals that the bulk of the features is unsuitable for the implementations. As a result, identifying significant features associated with LBW is an essential initial step. An assessment of the literature [4, 22] and data availability is used to do this. These 25 features are shown in Table 1 below.

**Table 1:** Features identified for low birth weight classification

No.	Variable name	Variable label
1	Residence	Type of place of residence (Urban/ Rural)
2	Education	educational level (no education/primary/secondary or higher)
3	Iron	Taking iron pills, sprinkles or syrup (No/ Yes)
4	Wealth	wealth index combined (Poorest/poorer/middle/richer/richest)
5	BMI	Body mass index (numerical)
6	Agem	Women's age in years (numerical)
7	Anaemia	Anemia level (severe/ moderate /mild/not anemic)
8	Orderbirth	Birth order number (numerical)
9	Twin	The child is a twin (single/multiple)
10	Gender	Sex of child (male/female)
11	Visits	Number of antenatal visits during pregnancy (numerical)
12	Delivery	Delivery by caesarean section (no/yes)
13	Smoking	Smokes cigarettes (no/yes)
14	Insurance	Covered by health insurance (no/yes)
15	Occuptiion	Occupation (No/Yes)
16	Sex of child	Sex of child (Male/Female)
17	Ethnicity	Ethnicity (Amahara/Oromo/Tigrie/Somali/Guragie/Others
18	Parity	Parity lab (1/2/3/4/5 +)
19	BTI	Birth Interval (Numeric)
20	Marital	Current marital status
21	Religion	Religion(orthodox/muslim/protestant/others)
22	Region	Region (11 categories)
23	Desirability	Desirability of the pregnancy (then/later/no more)
24	Sign in ANC	Sign Complexity during Antenatal care visit(s) (No/Yes)
25	Nutritional	Nutritional counseling (No/Yes)

Relevant features determine the performance of any machine learning model implementation. Feature selection refers to picking critical characteristics for the research work at hand. In addition to lowering data dimensionality, it improves data visualization and understanding. If further study is done, the effects of all 25 qualities must be considered. The topic of categorical data discretization is widely covered in [22]. An imbalanced dataset was resampled before training the prediction model, which may be considered a data preprocessing step. For resampling imbalanced datasets, SMOTE proven to nearly continuously improve classification performance [21].

### Machine learning classifiers

Some researchers have used machine learning approaches to anticipate medical problems and other disorders [22, 23]. Random forests, support vector machines, logistic regression, decision trees, and other approaches are among them. XGB (Extreme Gradient Boosting) is a widely utilized and efficient machine learning technique with a surprising impact. As a result, this research offered a correlation-based feature selection method to understand better what medical features may influence low birth weight outcomes. On the other hand, this study used a novel integrated learning algorithm called XGB to efficiently handle vast amounts of medical data and fulfil real-world requirements [20, 23]. As a result, the study's technique is innovative, combining theory and practice.

Because of its ability to predict and ease usage, Extreme Gradient Boosting (XGB) has become one of the most popular machine learning approaches. The regression and classification algorithms are being monitored. The two principles that make up XGB are decision trees and gradient boosting. Decision trees are likely the most easily interpretable machine learning algorithms known, and they may be pretty powerful when used in conjunction with the correct techniques. The nodes and leaves of a decision tree resemble the nodes and leaves of a tree with a base and numerous branches [23].

XGB is a boosting method that is part of an ensemble learning algorithm. The goal of XGB is to keep adding trees and performing feature splitting to expand a tree. When a tree is added, it learns a new function to suit the residuals of the previous prediction. The score of a sample is predicted when k trees are created after training. In reality, each tree will have a matching leaf node based on the features of this sample, and each leaf node correlates to a score. The sum of the scores for each tree equals the sample's predicted value. XGB adds a regular term to the loss function's second-order Taylor expansion to balance the model's complexity and the loss function's decline. It looks for the optimal solution globally and prevents model overfitting to a large extent.

K-Nearest Neighbor (K-NN) method selects the K samples with the most significant sample similarity to be graded, the voting approach determines which group the samples belong to, and the instance is divided into this class. The K-NN classification algorithm performs poorly in an adversarial environment. It is simple to use and maybe put to good use in some circumstances [18].

The Random Forest (RF) classifier has addressed classification and regression problems. Each split decision employs the notion of multiple random tree creation, with training bootstrap, sample bagging, voting system, and randomly picked characteristics. It takes substantially less input preparation, uses many types of features without normalization, and is quick to train and optimize according to hyperparameters [19].

**Fig. 1:**
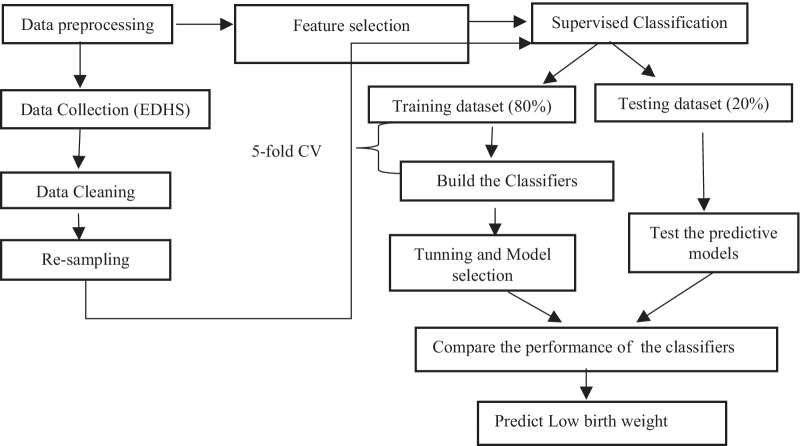
Flow chart of predictive classification

The LBW outcomes prediction model can be created and Logistic Regression (LR), Decision Tree (DT), Naive Bayes (NB), K-Nearest Neighbor (K-NN), Random Forest (RF), Support Vector Machine (SVM), Gradient Boosting (GB), and Extreme Gradient Boosting (XGB) [18, 20, 23, 24]approaches outlined above. Figure 1 depicts the prediction model flowchart. Following comparison and verification, the best feature combination is identified and utilized as the input to the final model prediction model [23].

### Hyperparameters tuning and evaluation

Hyperparameters describe the depiction architecture, and hyperparameter tuning is the process of optimizing model design. These approaches demonstrate how to use the space of potential hyperparameter values to describe likely model structures. This study employed Randomized Search cross-validation to improve the parameters of Logistic Regression (LR), Decision Tree (DT), Naive Bayes (NB), K-Nearest Neighbor (K-NN), Random Forest (RF), Support Vector Machine (SVM), Gradient Boosting (GB), and Extreme Gradient Boosting (XGB) [18–20, 23, 24].

Evaluation metrics are used to assess the performance and effectiveness of the implemented predictive model [24]. Their short descriptions are as follows: confusion matrix is a table that allows visualization of the performance of a supervised learning algorithm. The uncertainty matrix is deceptively easy to understand, but the associated words can be perplexing. True positives (TP) refer to the correctly classified samples in their correct class. True Negatives (TN) refer to the correctly classified samples that do not belong to the target class. False Positives (FP) refers to the samples incorrectly labelled as the target class when they are not. False Negatives (TN) refer to the samples incorrectly labelled as not the target class when they are [24].

Accuracy measures how many of the cases are correctly identified/predicted by the model [24], i.e. correct prediction divided by the total sample size; Recall or Sensitivity measures the rate of true positives, how many of the actual positive cases are identified/predicted as positive by the model; Precision measures how many of the positive predicted cases are actually positive; F1-Score combines the precision and recall of the model and it is defined as the harmonic mean of the model’s precision and recall; Area Under Curve (AUC) is the Area under the receiver operating characteristic curve and provides a comprehensive assessment of the accuracy of a model by screening the range of threshold values for the decision making; ROC curves is a receiver operating characteristic (ROC) curve illustrates the performance of a binary classification algorithm as a function of Ture positive rate and false positive rate; Precision-Recall tradeoff (AP) calculates the Area Under the Precision-Recall Curve to get one number that describes model performance; Area under Receiver operating characteristics curve (AUROC) makes use of True Positive Rates (TPR) and False Positive Rates (FPR) [24].

## Results

### Exploratory data analysis (EDA)

**Fig. 2:**
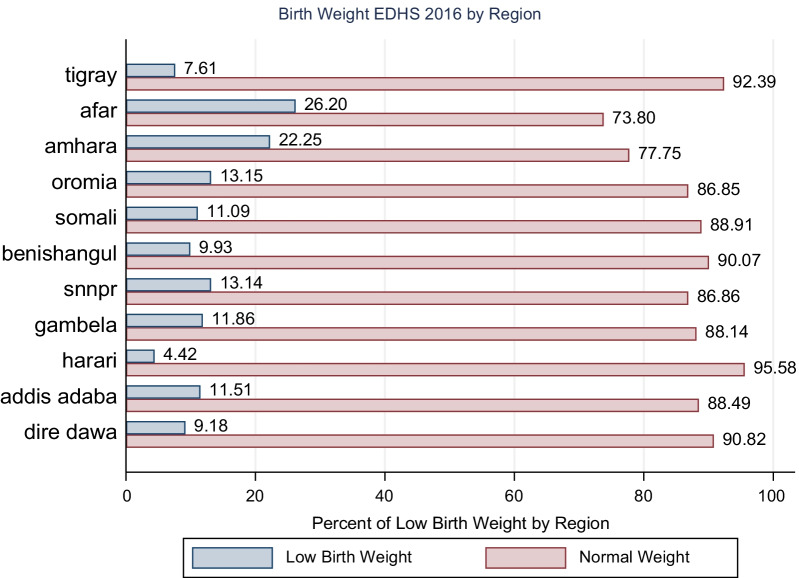
Birth weight by Region, Ethiopia

The total count of the sample is 2110. As shown in Table 2, the average age of mothers in the sample is 28 years, the mother's oldest is 47 years, whereas the youngest mother is 16 years old. The average registered body mass index is 22.445, the maximum index is 39.15, and the minimum index is 1358. Antenatal visit: 5 mean, 20 maximum and 0 minimum. The average birth order registered is 2.8, with a standard deviation of 2.1 and a maximum of 13. The average recorded birth weight is 3268.983 g, the maximum weight is 6000, the minimum weight is 500, and quartiles one and three were 3000 and 3750, respectively. Table 3 presented the cross-tabulation of target variable with its features. The continuous features are somewhat normally distributed, and there are many outliers present. Preprocessing on the data conducted to remove the irregularities. One-Hot Encoding on features conducted for the binary categorical features, and dummy Encoding on features employed for more than two categorical features. Thus, there are 2110 instances and 25 features and then the encoded features became 49. Figure 2 depicted the birth categories over the region in Ethiopia. The supplementary documents included additional EDA results in Additional file 1: Figs. S1–S5.

**Table 2:** Numerical attributes summary statistics

Variable	Mean	SD	Min	Q1	Q2	Q3	Max
BMI	22.445	4.32.816	1358	19.23.3	21.42	24.94	39.15
Age	28.396	6.046	16	24	28	32	47
Visits	4.679	2.311	0	4	4	5	20
Birth Order	2.769	2.121	1	1	2	4	13
Birth weight	3268.983	824.046	500	3000	3010	3750	6000

Q1, Q2, and Q3 are quartiles

**Table 3:** Cross-tabulation Birth Weight Category and Features

	LBW %	NBW %		LBW %	NBW %
*Delivery by caesarean section*			*Taking Iron Pills, Sprinkles Or Syrup*		
No	13.5	86.5	No	14.2	85.8
Yes	9.5	90.5	Yes	5.8	94.2
*Residence*			*Occupation*		
Urban	10.9	89.1	Not Working	13.3	86.7
Rural	15.4	84.6	Working	13.1	86.9
*Smokes Cigarettes*			*Sex Of Child*		
No	13.2	86.8	Male	10.9	89.1
Yes	9.6	90.4	Female	15.6	84.4
*Health Insurance*			*Child Is Twin*		
No	13.2	86.8	Single Birth	12.3	87.7
Yes	13.8	86.2	Multiple Birth	39.9	60.1
*Under Age 18 (Mothers’)*			*Current Marital Status*		
Under Age 18	44.9	55.1	Not Married	10.8	89.2
Age 18 Or Older	13	87	Married	13.4	86.6
*Sign Of Complexity During ANC*			*Nutritional Counseling*		
No	13.9	86.1	No	15.1	84.9
Yes	12.5	87.5	Yes	12.1	87.9
*Educational Level*			*Wealth Index Combined*		
No Education	18.3	81.7	Poor	15.7	84.3
Primary	11	89	Middle	17.3	82.7
Secondary > =	11.3	88.7	Rich	11.7	88.3
*Anemia Level*			*Desirability Of The Pregnancy*		
Severe	53.9	46.1	Then	14	86
Moderate	13.6	86.4	Later	9.9	90.1
Mild	16.7	83.3	No More	8.7	91.3
Not Anemic	12.4	87.6			
*Religion*			*Ethnicity*		
Orthodox	13.4	86.6	Amhara	15.8	84.2
Muslim	15.8	84.2	Oromo	16.3	83.7
Protestant	9.6	90.4	Tigrie	6.5	93.5
Others	1.6	98.4	Somalie	10	90
			Guragie	16.7	83.3
			Other	10.4	89.6
*Ethnicity*			*Parity*		
Amhara	15.8	84.2	1	9.8	90.2
Oromo	16.3	83.7	2	13.1	86.9
Tigrie	6.5	93.5	3	17.5	82.5
Somali	10	90	4	18.7	81.3
Guragie	16.7	83.3	5 +	12.4	87.6
Other	10.4	89.6			

LBW denotes Low Birth Weight; NBW denotes Normal Birth Weight

**Table 4:**
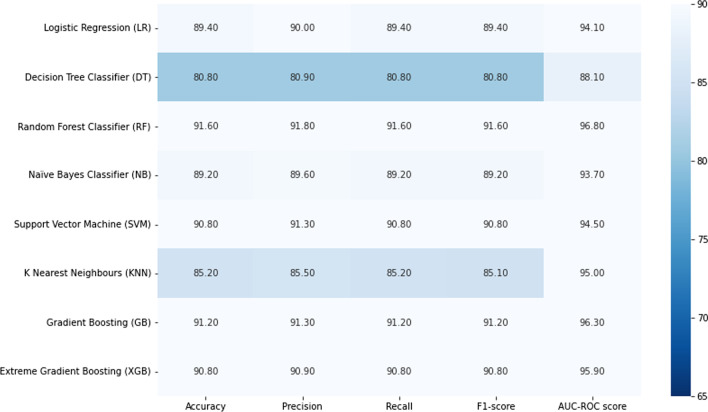
Performance metrics of the classifiers

### Data Preprocessing

Through data processing, the number of samples in the data set is 2110 instance and 25 features. The LBW and Normal weight samples ratio is 1:9, which belongs to the category imbalanced data set. The study dataset resampled to improve the imbalance ratio. This study employed the SMOTE method [21].

### Implementation LBW prediction models

Once the preprocessing is done, the implementation of predictive models can commence. Numerous combinations of parametric values and settings are carried out. The model is fine-tuned to get optimal performance. These settings are divided into four categories general, booster, learning task, and command line. Tuning can be done in a grid or by searching randomly. The grid search is used in this study. With many factors, grid search for the best solution might be challenging. It can be readily addressed by focusing on a smaller number of parameters with suitable parameter ranges. K-fold cross-validation is used during the model selection step to assess model performance. Packages and libraries of Python utilized. The grid search is done in the following manner.

First, to check for over-fitting, the “n estimators” that define the model's epoch are set to 100 and early stopping rounds to 5. Second, find the best learning rate and gamma at the same time, as they have a direct impact on the model's performance. 0.01, 0.02, 0.03, 0.06, 0.1, 0.2, and 0.3 are the grid values for the learning rate, whereas 0, 0.1, 0.2, 0.5, and 1 are the grid values for the gamma. For model tuning, all potential combinations of these two parameter values are tested, and the ones that perform best are kept as the optimal values. Third, make a grid search across the max depth and min child weight in the ranges of 1–10 using the optimal learning rate values and gamma. Fourth, make a grid search across the regularization parameter reg lambda and subsample in the 0.1–1 range simultaneously. Fifth, re-examine the model using a simultaneous grid search over gamma, reg lambda, and subsample to check for discrepancies between the optimal values. This study divided the data into a training set and test set account 80% and 20%, respectively. The model performance, such as prediction evaluation metrics, can be compared against different ML algorithms and parameter settings. This study used the popular fivefold cross-validation to avoid overfitting.

**Fig. 3:**
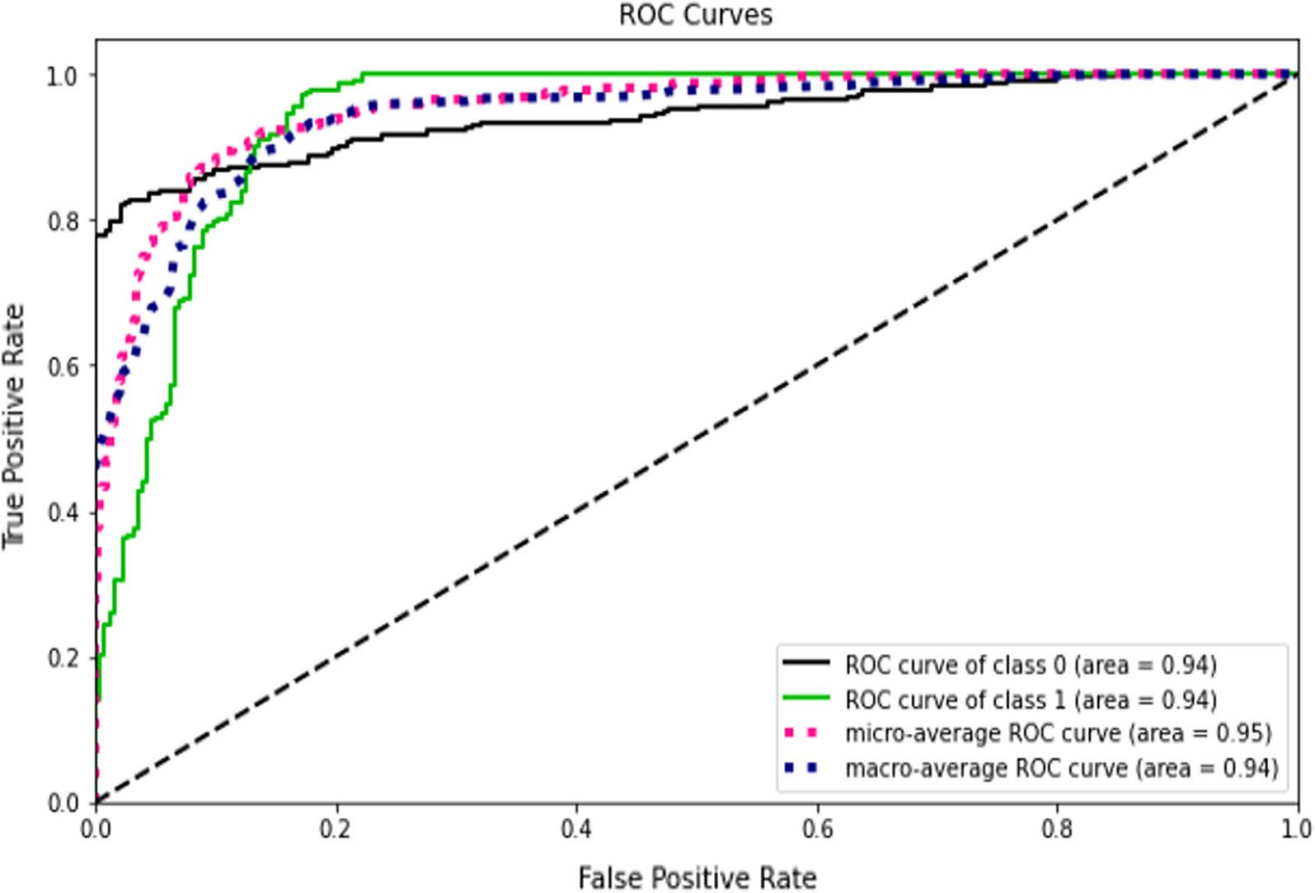
Logistic regression

**Fig. 4:**
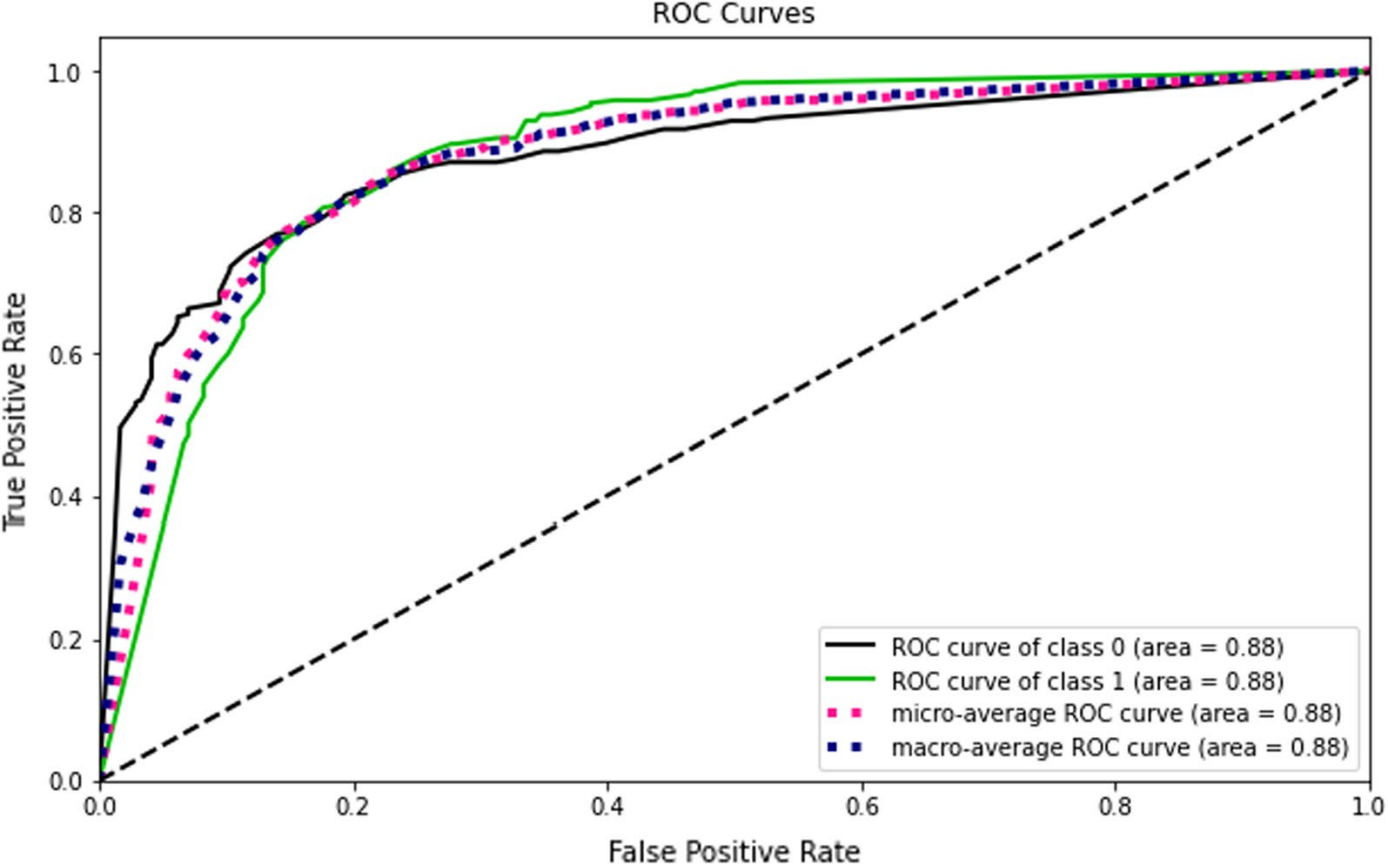
Decision tree

**Fig. 5:**
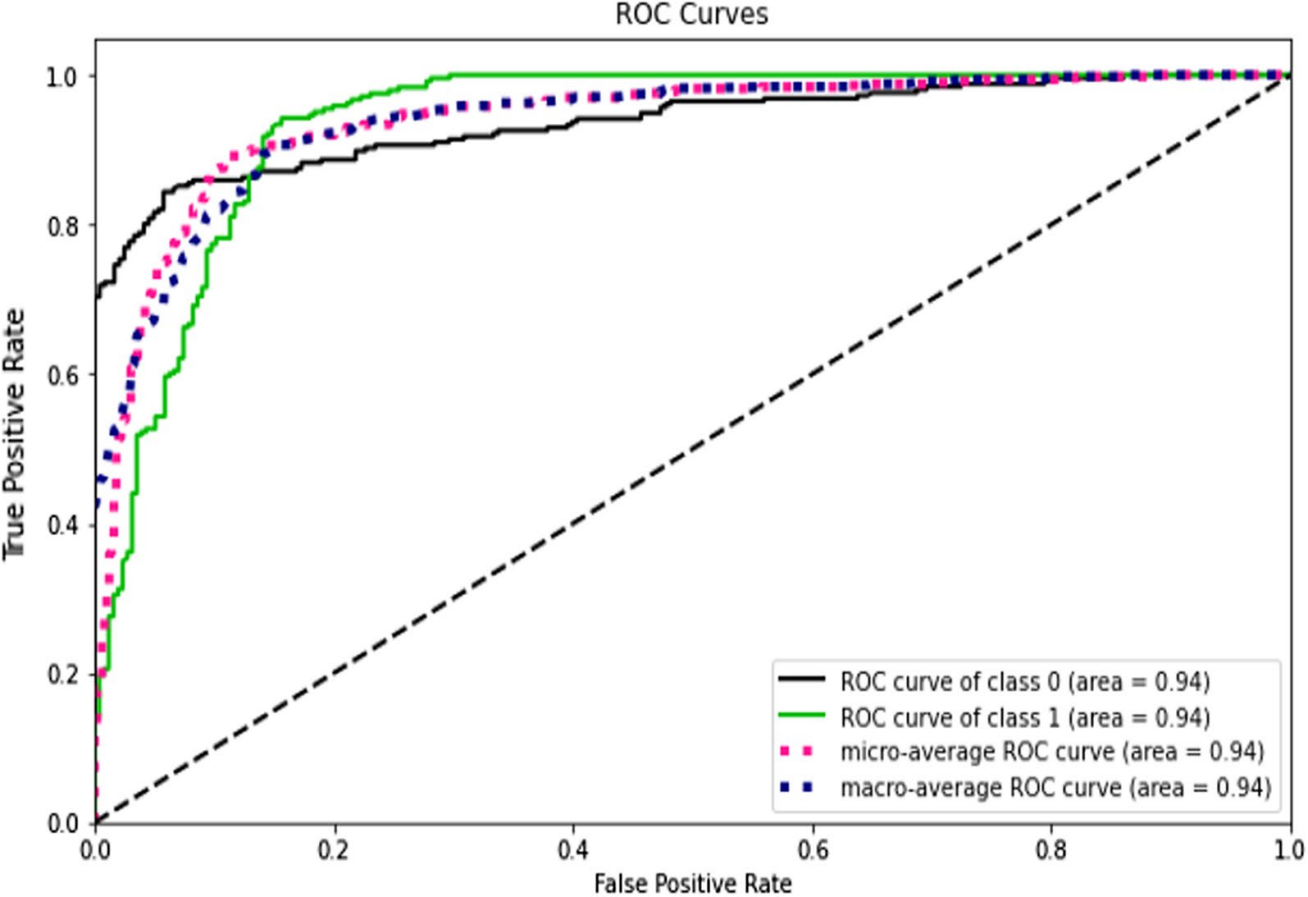
Naives bayes

**Fig. 6:**
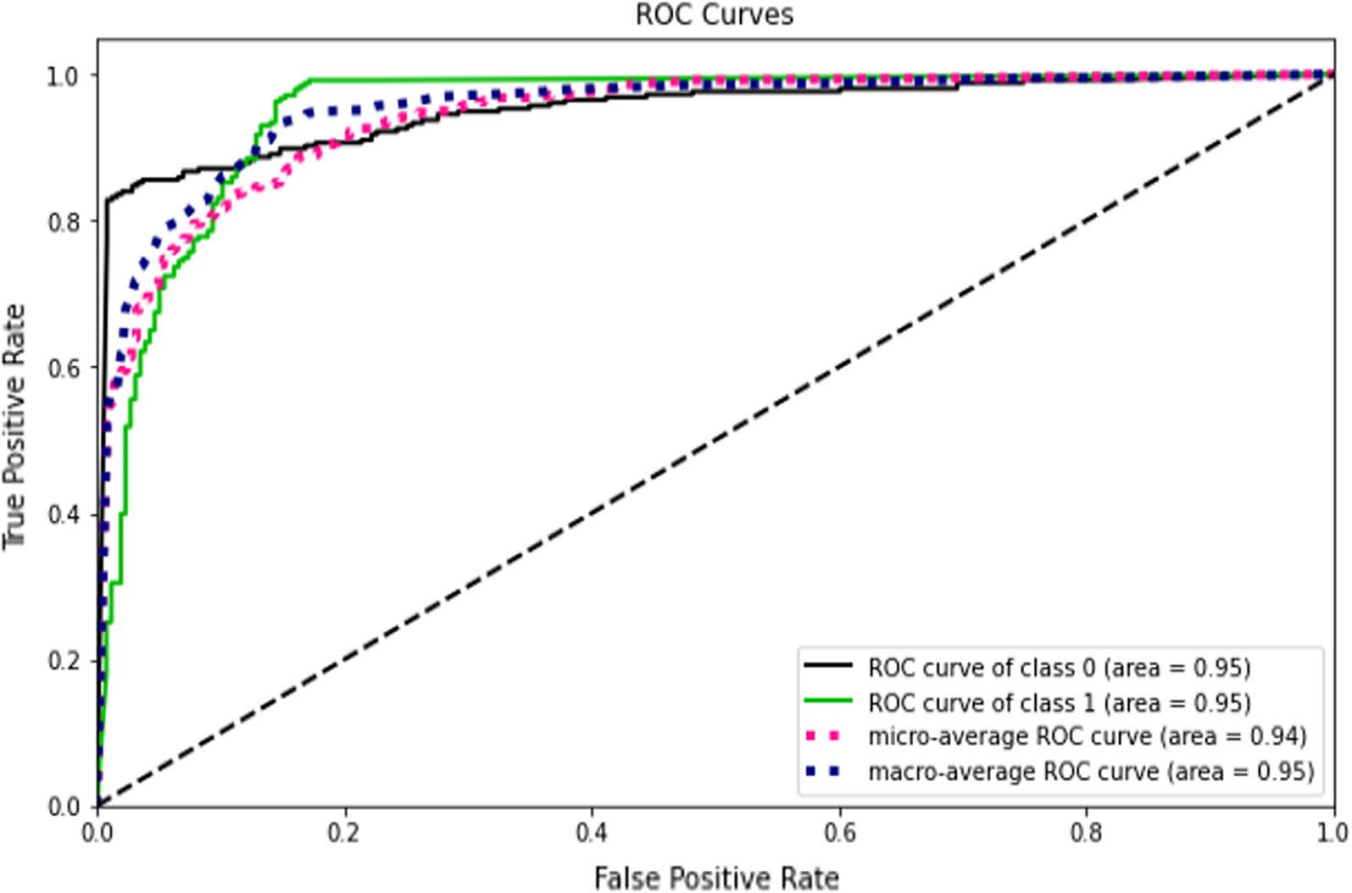
KNN

**Fig. 7:**
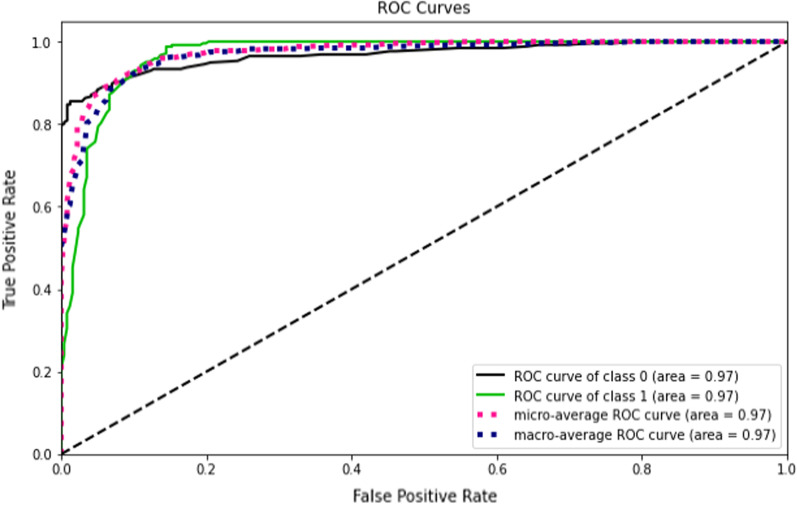
Random forest (RF)

**Fig. 8:**
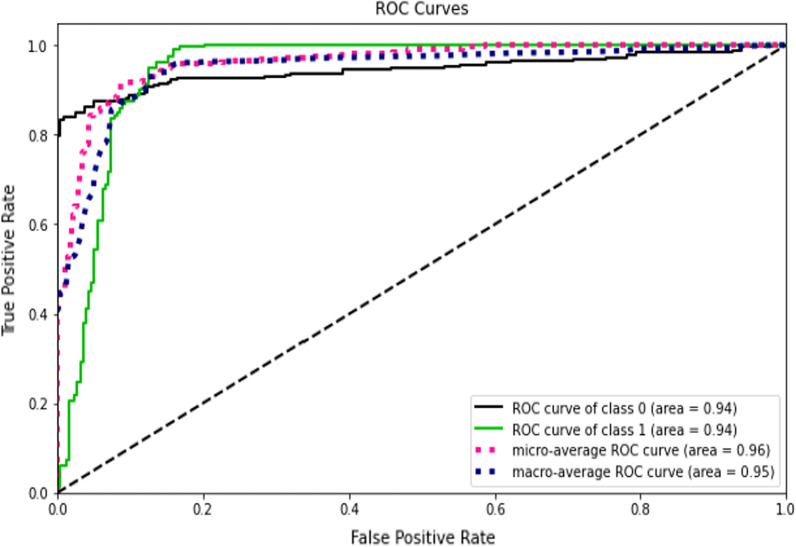
SVM

**Fig. 9:**
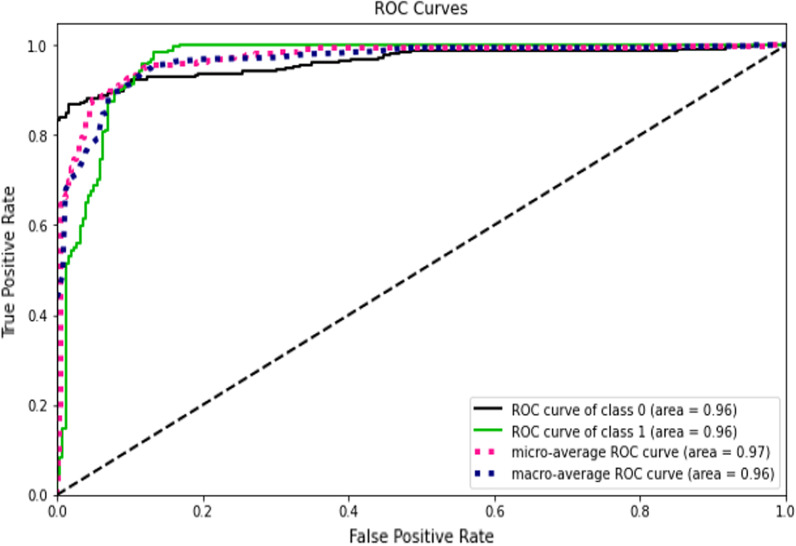
Gradient boost

**Fig. 10:**
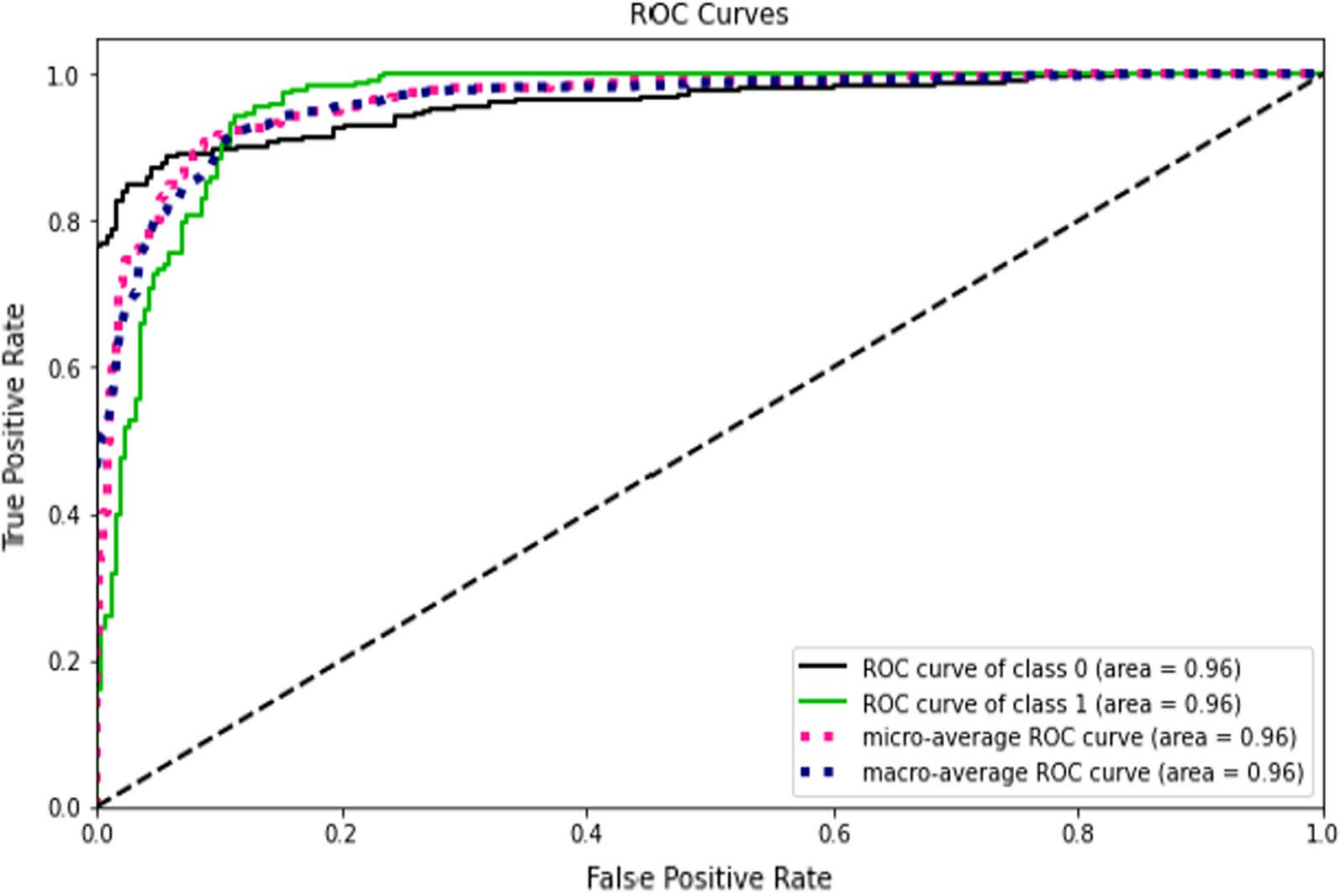
Extream Grient boost

**Fig. 11:**
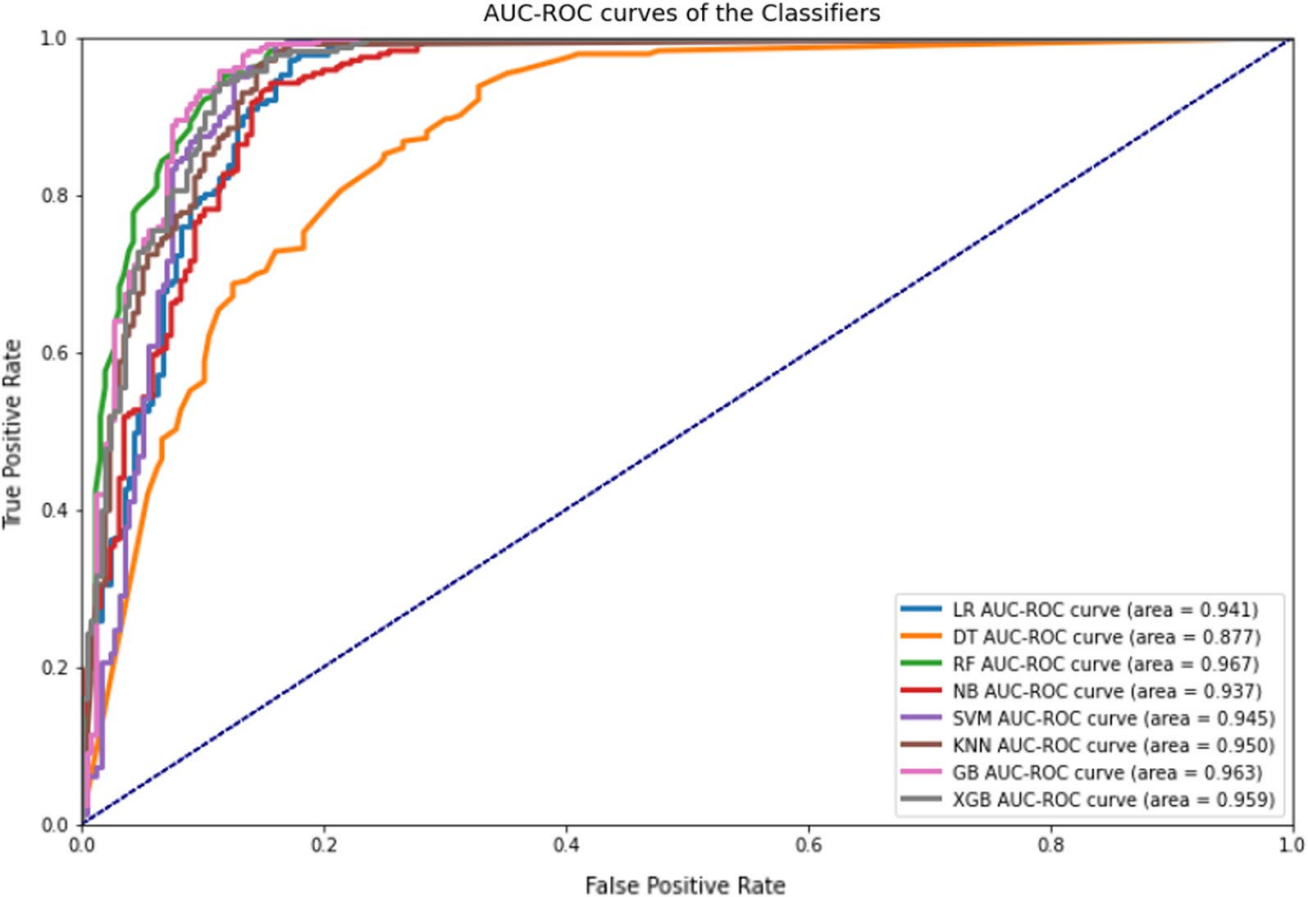
AUC-ROC curves of the classifiers

This study compared the eight different classification methods to verify the superiority of one of the proposed methods. The LBW outcome prediction of Logistic Regression (LR) (Fig. 3), Decisoin Tree (DT) (Fig. 4), Naive Bayes (NB) (Fig. 5), K-Nearest Neighbor (K-NN) (Fig. 6), Random Forest (RF) (Fig. 7), Support Vector Machine (SVM) (Fig. 8), Gradient Boosting (GB) (Fig. 9), and Extreme Gradient Boosting (XGB) (Fig. 10) method were depicted. The models are trained and tuned on the training dataset and compared the ML classifiers on the training prediction model. The model performance evaluation criteria are accuracy, AUC, F1, AP, ROC_AUC, and recall. Overall, RF has the best performance, and thus it was taken forward as the best ML algorithm. The results are shown in Table 4 and Fig. 11.

In this study, the RF model is the best classifier with all performance metrics and predicts LBW. 96.01% of average Precision implies the tradeoff between Precision and recall and computes the Area Under the Precision-Recall Curve. 89.72% of accuracy indicates the amount of correctly predicted cases in the XGB of LBW. 96.13% of Sensitivity or recall implies actual LBW cases predicted as LBW by the classifier. F1-Score implied result of the Precision and recall of the model. Area Under Curve (AUC) was 90.01%, which provides a comprehensive assessment of the accuracy of a model by screening the range of threshold values for decision making.

As Table 4 presented, the percentage of normal and low birth weight targets that are classified is as low or normal birth weight. The hamming loss has the best value of 0 and the worst value of 1. The RF study's Hamming loss result is 0.103, which is practically the best of misclassification.

Furthermore, the Jaccard score is the ratio of the size of the intersection to the size of the union of predicted and ground truth label classes.

When combining predicted and actual classes, it is called a similarity coefficient. The best classification is one, and the poorest rating is 0. This study’s Jaccard score is 0.819, implying that the actual labels are best predicted.

**Fig. 12:**
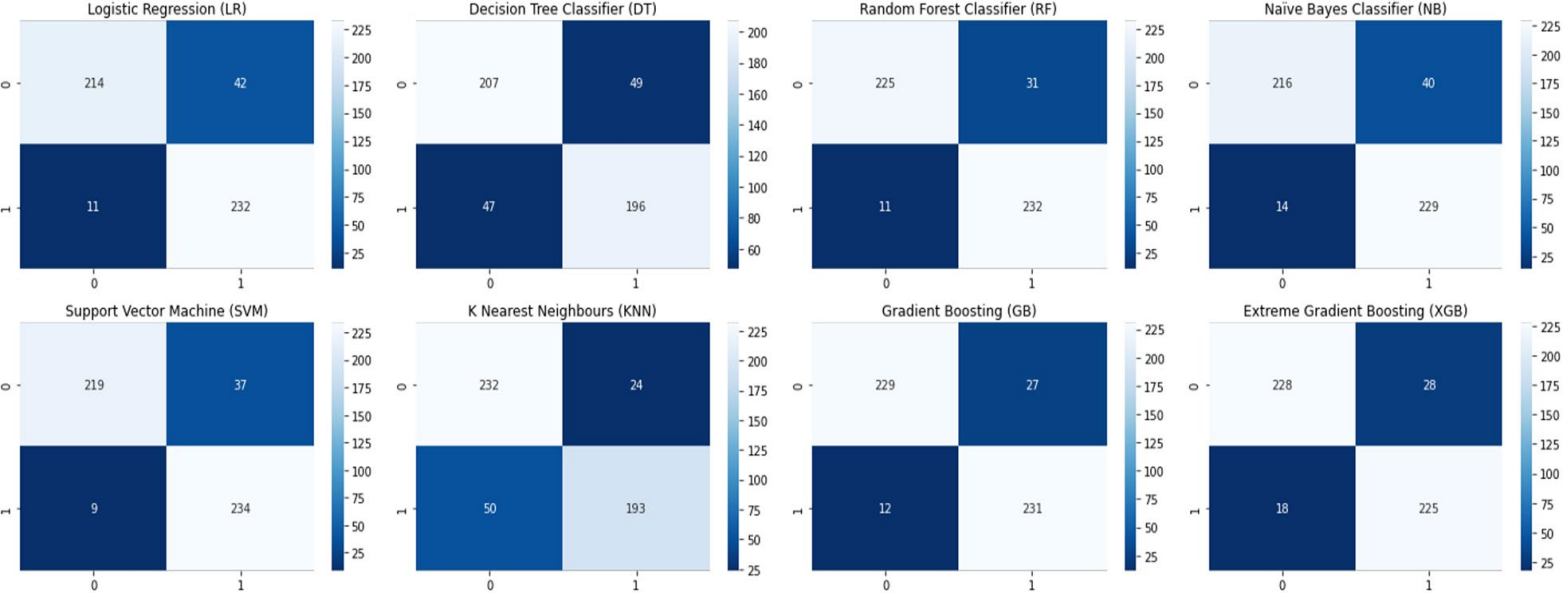
Confusion matrix of the classifiers’

Overall, the RF classifier outperforms the others. It is not proof that RF is always better than other algorithms, as the nature of the data might significantly impact which algorithms are employed. It could be argued that, among the most common tools, RF is the best tool for implementing a predictive model employing the EDHS. The confusion matrix is shown in Fig. 12. Note that the numbers in the confusion matrix had been used to calculate the performance measures shown in Table 4.

**Fig. 13:**
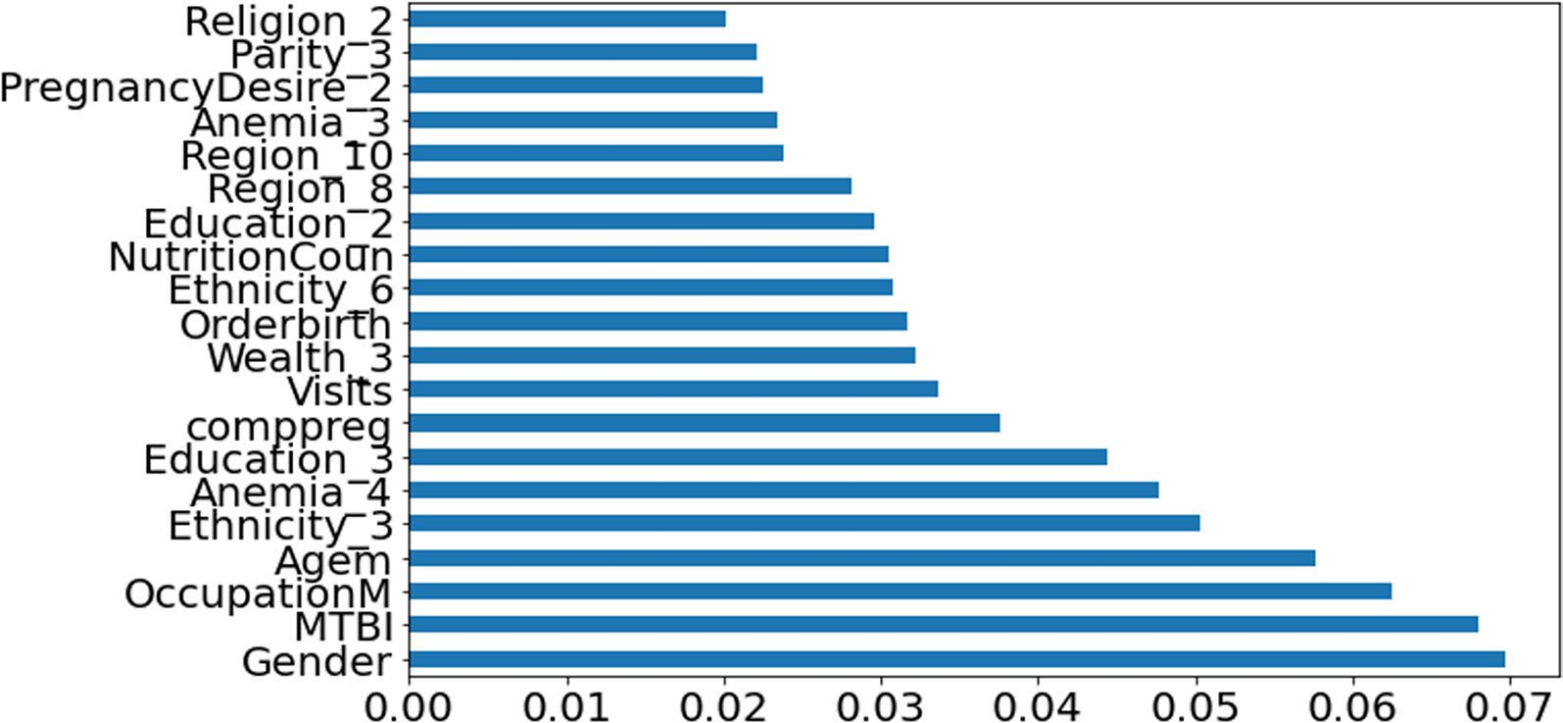
Feature importance of the RF in the prediction classification

Finally, RF classification of LBW, the results of an attribute in all the promotion trees are weighted and summed and then averaged to obtain the importance score. The  RF model provides an order of important feature for enhancing the accuracy of the prediction model while performing low birth weight prediction using the tree model method. As It is shown in Fig. 13.

According to previous research [1, 15, 16], Ethiopia is one of the countries with the most significant number of low birth weight babies globally. Furthermore, while the prevalence of low birth weight has decreased in the country from time to time, more work is needed to promote this decline and to limit the detrimental implications of the occurrence [1, 4, 13, 25]. In this study, the average birth weight was 3268.983 g, with a maximum of 6000 g and a minimum of 500 g, while quartiles one and three were 3000 and 3750 g, respectively. The sample's mothers are 28 years old, with the oldest being 47 years old and the youngest being 16 years old. The average registered BMI is 22.445, with a maximum index of 39.15 and a low of 1358. Antenatal visits: 5 on average, 20 on average, and 0 on average. With a standard deviation of 2.1 and a maximum of 13, the average birth order is 2.8. The classification machine learning method compared, identified, and helped recognize specific attributes related to low birth weight in Ethiopia that can be used as intervention targets. When compared to other machine learning classifier models such as the RF and KNN, the Extreme Gradient Boosting (XGB) model has the highest prediction power among the predictive models built.

As the feature importance rank identified, Gender of the child, marriage to birth interval, mother’s occupation and mother's age were the top four critical predictors of low birth weight in Ethiopia, according to the XGB model. This study is roughly in line with the findings research [6, 10, 14, 16, 22]. The findings of this study are similar to those of the subsequent analyses. The following factors were associated with nearby low birth weight: region, religion, residency, birth type, birth preparedness, fast and rapid breathing, maternal education, and maternal age [26]. The first important feature of Ethiopia's predictors of low birth weight was the child's gender. According to research conducted in Ethiopian hospitals, female newborns were more likely to develop LBW [27].

According to a 2011 EDHS study, there is a link between maternal education and proxy LBW [28]. Multiple births were more likely to result in proxy LBW during the first and second parties than single births [29]. Research from Addis Ababa hospitals, capital of Ethiopia, on the other hand, found a link between proxy LBW and birth preparedness. According to the study, planned birth reduces the risk of proxy LBW by 70% compared to uncontrolled delivery [30]. When a pregnancy is unplanned, socioeconomic, psychological, behavioral, or, job-related concerns may be used to justify having LBW.

The mother's age was one of the most critical indicators of low birth weight in Ethiopia in this study. In Ethiopia, a mother's age increases the likelihood of a low birth weight baby. The similarities between these studies might be due to mothers' physiological, psychological, and behavioral immaturity in this age range, which affects their children's ability to provide enough nourishment and care. Maternal education was linked to proxy LBW [31]. This study also discovered that Ethiopia's region was one of the most critical indicators of low birth weight. Another study found that neonates born in the Gambella and Somalia regions had a lower risk of becoming proxy LBW [28]. Maternal factors are probably to blame. Mothers from the Afar area may be more stunted or undernourished than those from Dire-Dawa, and vice versa for Somalia and Benishangul Gumz. Furthermore, the socioeconomic status may have a role in having low birth weight infants. For instance, Afar, Somalia, and the SNNPR have a lower socioeconomic level than Dire-Dawa [17].

Neonates born in rural areas have a higher risk of proxy LBW than those born in urban areas [28]. However, the current study's findings contradict a study done in Dire-Dawa, which found no link [32]. Many couples prefer males and offer appropriate care for boys rather than females during conception. A desired later birth has a higher risk of proxy LBW than a planned birth [15].

## Conclusions

Infant mortality and its implications have been linked to low birth weight. Predicting the LBW is thus a valuable preventative tool and predictor of newborn health hazards. This study used the Ethiopia Demographic and Health Survey 2016 to develop predictive LBW models. This study used Logistic Regression (LR), Decision Tree (DT), Naive Bayes (NB), K-Nearest Neighbor (K-NN), Random Forest (RF), Support Vector Machine (SVM), Gradient Boosting (GB), and Extreme Gradient Boosting (XGB) to compare and find the best classifier for predictive classification. Before applying the predictive models, data preprocessing is carried out, including data cleaning. In this study, the classifier categories are normal and LBW. RF was the best classifier, predicting LBW with 91.60 percent accuracy, 91.60 percent Recall, 96.80 percent ROC-AUC, 91.60 percent F1 Score, 1.05 percent Hamming loss, and 81.86 percent Jaccard score, according to the research. As a result, the RF predicts the occurrence of LBW correctly and more effectively than other classifiers. Gender of the child, marriage to birth interval, mother’s occupation and mother’s age were Ethiopia's top four critical predictors of low birth weight. This study has a direct importance on Ethiopian health and preventative policymaking related with low birth weight.

## Supplementary Information

**Additional file 1.** Explanatory Data Analysis of Low birth Weight in Ethiopia based on EDHS 2016.

## Data Availability

The datasets generated and/or analyzed during the current study are available in http://dhsprogram.com/data/available-datasets.cfm.

## Abbreviations

AUC: Area under the curve
EDHS: Ethiopian demographic and health survey
BMI: Body mass index
RF: Random forest
ROC: Receiver operating characteristic
LBW: Low birth weight
K-NN: K-nearest neighbor
XGB: Extreme gradient boosting
