# Supplementary material for: Machine learning algorithms for predicting low birth weight in Ethiopia
Source: BMC Med Inform Decis Mak. 2022 Sep 5;22:232. doi: 10.1186/s12911-022-01981-9 (PMC9443037; doi:10.1186/s12911-022-01981-9)
Supplement: Supplementary file 1 — Additional file 1. Explanatory Data Analysis of Low birth Weight in Ethiopia based on EDHS 2016. [file 12911_2022_1981_MOESM1_ESM.docx]

**Explanatory Data Analysis of Low birth Weight in Ethiopia based on EDHS 2016**


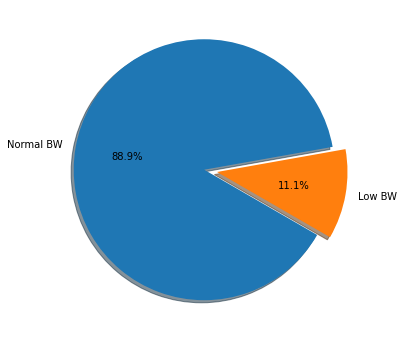


Figure S1 Pie-chart for Birth Weight Normal and Low birth weight

The Target Variable seems imbalanced.


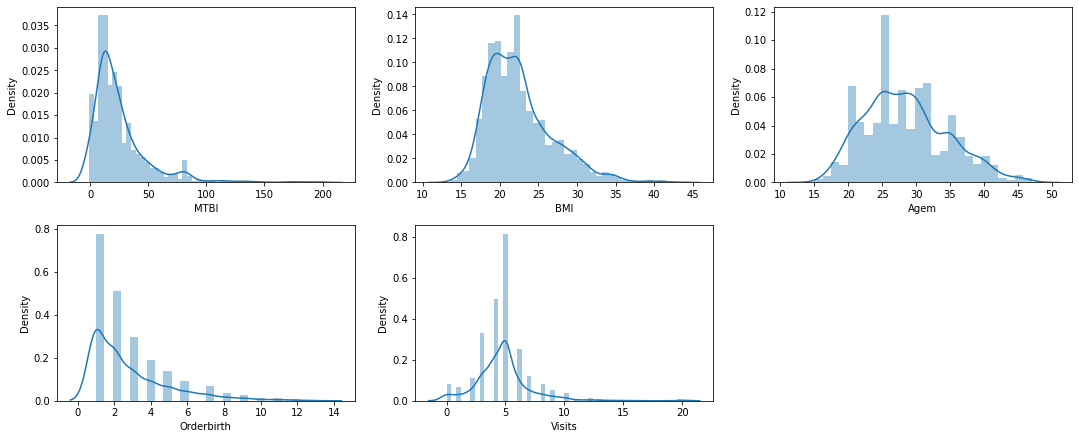


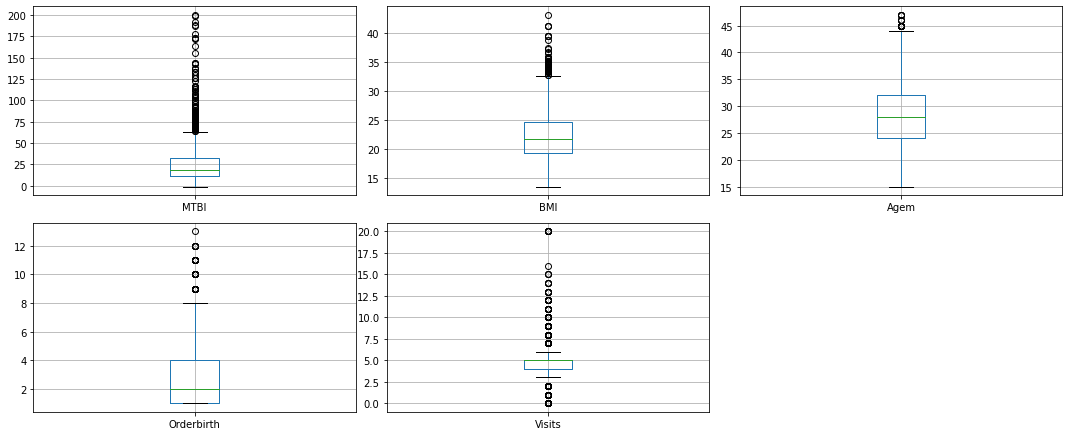


Figure S2 Continuous features: density and box plots

The continuous features are somewhat normally distributed, and there are many outliers present. Preprocessing on the data conducted to remove the irregularities.


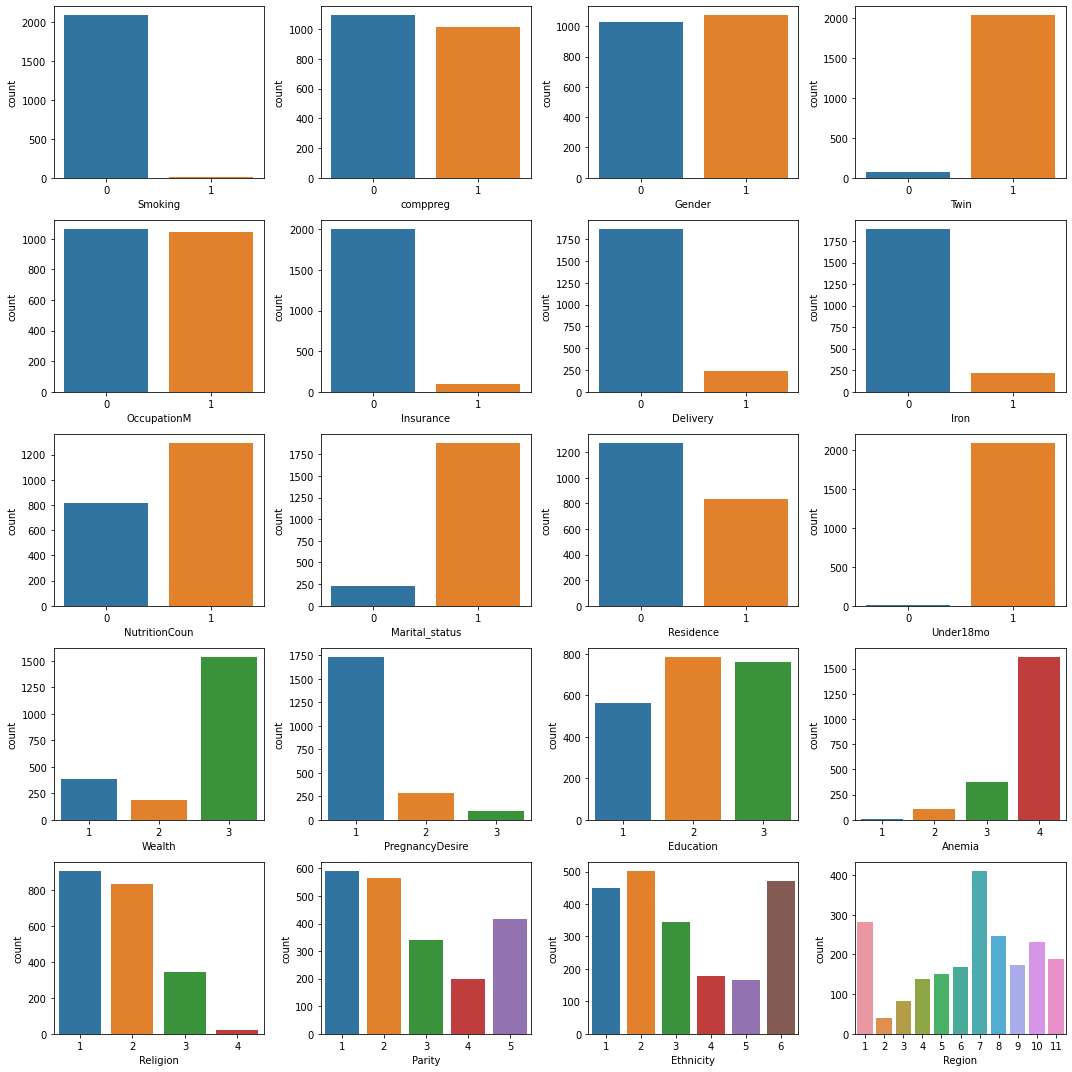


Figure S3 Categorical features bar plots

One-Hot Encoding on features conducted for the binary categorical features: Smoking, comppreg, Gender, Twin, OccupationM, Insurance, Delivery, Iron, NutritionCoun, Marital_status, Residence, Under18mo

Dummy Encoding on features employed for more than two categorical features: Wealth, PregnancyDesire, Education, Anemia , Religion, Parity, Ethnicity, and Region

There are 2110 instances and 25 features and then the encoded features became 49.


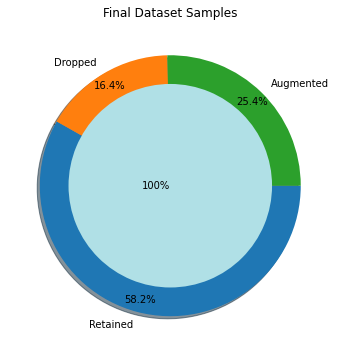


Figure S4 Final (Processed) Dataset Plots

- After removal of outliers, the dataset now has 1406 samples.
- The Target Variable seems imbalanced. The imbalance fixed using SMOTE Technique.
- The final dataset after cleanup has 2492 samples and 49 columns.
- Splitting the data intro training & testing sets

Original set ---> (2492, 48) (2492,)

Training set ---> (1993, 48) (1993,)

Testing set ---> (499, 48) (499,)

- Feature Scaling (Standardization) by StandardScaler()


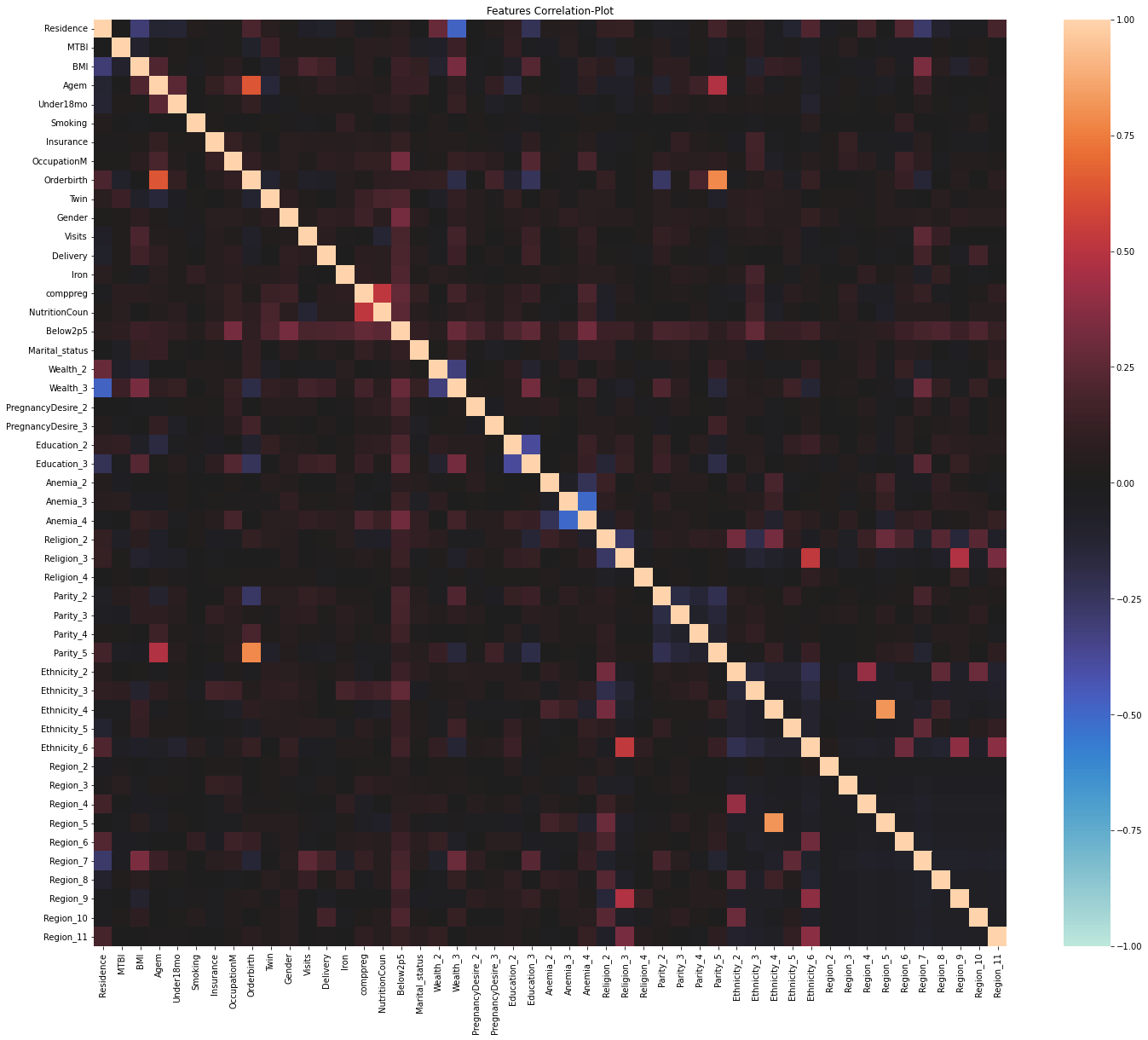


Figure S5 Features Correlation Heat Plot

|  | Birth Weight </>= 2.5kg | | |
| --- | --- | --- | --- |
|  | Low BW % | Norma BW % | Total |
|  | % | % | % |
| delivery by caesarean section | | |  |
| no | 13.5 | 86.5 | 100 |
| yes | 9.5 | 90.5 | 100 |
| Total | 13.2 | 86.8 | 100 |
| RECODE of v106 (highest educational level) | | | |
| no education | 18.3 | 81.7 | 100 |
| primary | 11 | 89 | 100 |
| secondary or above | 11.3 | 88.7 | 100 |
| Total | 13.2 | 86.8 | 100 |
| taking iron pills, sprinkles or syrup | | | |
| no | 14.2 | 85.8 | 100 |
| yes | 5.8 | 94.2 | 100 |
| Total | 13.2 | 86.8 | 100 |
| respondent's occupation (grouped) | | | |
| not working | 13.3 | 86.7 | 100 |
| professional/technical/managerial | 13.1 | 86.9 | 100 |
| Total | 13.2 | 86.8 | 100 |
| type of place of residence | | |  |
| urban | 10.9 | 89.1 | 100 |
| rural | 15.4 | 84.6 | 100 |
| Total | 13.2 | 86.8 | 100 |
| smokes cigarettes | |  |  |
| no | 13.2 | 86.8 | 100 |
| yes | 9.6 | 90.4 | 100 |
| Total | 13.2 | 86.8 | 100 |
| sex of child | |  |  |
| male | 10.9 | 89.1 | 100 |
| female | 15.6 | 84.4 | 100 |
| Total | 13.2 | 86.8 | 100 |
| child is twin | |  |  |
| single birth | 12.3 | 87.7 | 100 |
| 1st of multiple | 39.9 | 60.1 | 100 |
| Total | 13.2 | 86.8 | 100 |
| RECODE of v190 (wealth index combined) | | | |
| poor | 15.7 | 84.3 | 100 |
| middle | 17.3 | 82.7 | 100 |
| rich | 11.7 | 88.3 | 100 |
| Total | 13.2 | 86.8 | 100 |
| ethnicity |  |  |  |
| amhara | 15.8 | 84.2 | 100 |
| oromo | 16.3 | 83.7 | 100 |
| tigrie | 6.5 | 93.5 | 100 |
| somalie | 10 | 90 | 100 |
| guragie | 16.7 | 83.3 | 100 |
| other | 10.4 | 89.6 | 100 |
| Total | 13.2 | 86.8 | 100 |
| covered by health insurance | | |  |
| no | 13.2 | 86.8 | 100 |
| yes | 13.8 | 86.2 | 100 |
| Total | 13.2 | 86.8 | 100 |
| under age 18 (from household questionnaire) | | | |
| under age 18 | 44.9 | 55.1 | 100 |
| age 18 or older | 13 | 87 | 100 |
| Total | 13.2 | 86.8 | 100 |
| anemia level | |  |  |
| severe | 53.9 | 46.1 | 100 |
| moderate | 13.6 | 86.4 | 100 |
| mild | 16.7 | 83.3 | 100 |
| not anemic | 12.4 | 87.6 | 100 |
| Total | 13.2 | 86.8 | 100 |
| Paritylab |  |  |  |
| 1 | 9.8 | 90.2 | 100 |
| 2 | 13.1 | 86.9 | 100 |
| 3 | 17.5 | 82.5 | 100 |
| 4 | 18.7 | 81.3 | 100 |
| 5+ | 12.4 | 87.6 | 100 |
| Total | 13.2 | 86.8 | 100 |
| RECODE of v501 (current marital status) | | | |
| Not married | 10.8 | 89.2 | 100 |
| Married | 13.4 | 86.6 | 100 |
| Total | 13.2 | 86.8 | 100 |
| RECODE of v130 (religion) | | |  |
| orthodox | 13.4 | 86.6 | 100 |
| muslim | 15.8 | 84.2 | 100 |
| protestant | 9.6 | 90.4 | 100 |
| others | 1.6 | 98.4 | 100 |
| Total | 13.2 | 86.8 | 100 |
| region |  |  |  |
| tigray | 7.6 | 92.4 | 100 |
| afar | 26.2 | 73.8 | 100 |
| amhara | 22.2 | 77.8 | 100 |
| oromia | 13.1 | 86.9 | 100 |
| somali | 11.1 | 88.9 | 100 |
| benishangul | 9.9 | 90.1 | 100 |
| snnpr | 13.1 | 86.9 | 100 |
| gambela | 11.9 | 88.1 | 100 |
| harari | 4.4 | 95.6 | 100 |
| addis adaba | 11.5 | 88.5 | 100 |
| dire dawa | 9.2 | 90.8 | 100 |
| Total | 13.2 | 86.8 | 100 |
| wanted pregnancy when became pregnant | | | |
| then | 14 | 86 | 100 |
| later | 9.9 | 90.1 | 100 |
| no more | 8.7 | 91.3 | 100 |
| Total | 13.2 | 86.8 | 100 |
| during (any of) your antenatal care visit(s), were you told about the signs of p | | | |
| no | 13.9 | 86.1 | 100 |
| yes | 12.5 | 87.5 | 100 |
| Total | 13.2 | 86.8 | 100 |
| did any health worker give you nutritional counseling? | | | |
| no | 15.1 | 84.9 | 100 |
| yes | 12.1 | 87.9 | 100 |
| Total | 13.2 | 86.8 | 100 |
